# Locking pegs versus locking screws in volar plating of distal radius fractures

**DOI:** 10.1007/s00068-025-02876-w

**Published:** 2025-05-22

**Authors:** Nicole M. van Veelen, Matija Horvat, Björn-Christian Link, Bryan J. M. van de Wall, Frank J. P. Beeres

**Affiliations:** 1 Department of Orthopaedic and Trauma Surgery, Luzerner Kantonsspital, Spitalstrasse 166000, Lucerne, Switzerland; 2 Faculty of Health Sciences and Medicine, University of Lucerne, Alpenquai 4, 6005 Lucerne, Switzerland

**Keywords:** Wrist fractures, Distal radius, Locking screw, Volar plate

## Abstract

**Purpose:**

The aim of this study was to compare the radiological outcome of patients with distal radius fractures stabilized with a volar plate using either locking screws or pegs.

**Material & Methods:**

For this retrospective study all adult patients that received volar plating of a distal radius fracture at a trauma center between 06/2019 and 06/2022 were eligible for inclusion. Only patients who received an implant allowing both locking pegs and screws were included. Primary outcome was radiological loss of reduction at the 6-week and at the 12-month follow-up. Secondary outcomes were duration of surgery, implant removal, fracture union and complications.

**Results:**

Fourty-nine patients treated with pegs and 39 with screws were included. Patient demographics were comparable, however there were more complex fractures in the peg group. There was no significant difference in the occurrence of radiological loss of reduction between the groups at 6 weeks or 12 months (p = 1). Patients treated with pegs were more frequently operated upon by experienced surgeons while screws were more often used by more junior staff. The duration of surgery was longer for patients who received screws (p = 0.003). Union was achieved in all fractures for which a 12-month x-ray was available. There was no significant difference in implant removal rate or other complications.

**Conclusions:**

Regarding secondary loss of reduction both locking pegs and screws show similar results. Considering the potential benefits of pegs, such as the smooth surface which may reduce the risk of joint penetration, pegs are a viable alternative to screws.

## Introduction

In western societies, distal radius fractures are the most frequently encountered fractures, typically affecting elderly women [1, 2]. This is due to the fact that distal radius fractures are associated with osteoporosis which affects more postmenopausal women than it does men [2–4]. While many distal radius fractures can be treated non-operatively, some require surgical treatment [4–7].

Over the last approximately eight decades operative techniques have evolved and improved. The development of fixed-angle plates has allowed for volar plating of dorsally unstable fractures by providing subchondral support. The benefits of volar over dorsal plating have been described previously and include minimizing tendon irritation and rupture [8]. The fixed-angle plates rely on neutralizing the forces leading to dorsal displacement by buttressing the subchondral bone of the distal fragment rather than actually fixating it with screws [8]. Therefore some implants allow for the use of smooth fixed-angle pegs or pins instead of screws. A possible benefit of the use of smooth pegs, is that due to their blunt tip, bicortical implants may be less likely to cause tendon irritation or rupture. Therefore, longer implants can be used which may improve stability. Another possible benefit of the smooth surface is a potentially lower risk of penetration into the joint in case of fractures subsidence.

Multiple biomechanical studies have been performed to evaluate the different fixation methods; however, no sound conclusion can be drawn as the results differ between the studies [9–12]. One clinical study with a small retrospective cohort was unable to show a significant difference with regards to radiological outcome between patients treated with locking screws and those treated with pegs [13].

The aim of this study was to compare the short and long-term radiological outcome of patients with distal radius fractures that were stabilized with a volar plate using either locking screws or locking pegs in the distal fragment. Secondary outcomes of interest included fracture union, duration of surgery, and implant removal.

## Material and methods

This study was written according to the STROBE-guidelines [14] and it was approved by the Swiss Association of Research Ethics Committees (PB_2016-01882).

### Study design, setting and participants

For this retrospective cohort study all patients with an acute distal radius fracture (AO type 2R3), that were treated with a volar plate at a level 1 trauma center in Switzerland between June 1 st 2019 and June 1 st 2022 were eligible for inclusion. A general consent form for the use of patient data for scientific purposes is obtained from all patients at the study hospital. The implant used, had to allow for both fixed angle screws and fixed angle pegs in the two distal screw rows (Titanium Volar Distal Radius Plating System 2.4/3.5 mm, Arthrex, Inc., Naples, FL, USA). Patients, who received more than a volar plate (for example additional plates, screws or k-wires) or those in which a combination of fixed angle screws and pegs were used in the distal two rows were excluded. A minimum follow-up of six weeks was required to be included in the study. Two groups were formed based on the implant used in the distal two rows of the volar plate, in one group only locking screws were used and in the other only locking pegs.

### Surgical technique and follow-up

All surgeries were performed by board certified surgeons or experienced trainees, who were supervised by board certified surgeons. Antibiotic prophylaxis was given according to the local guidelines (2 g cefazolin single shot 30 min prior to surgery). The patients were positioned supine with the arm extended on an arm table. A modified henry approach was used in all patients [5]. Whether screws or pegs were used in the distal two rows of the plate was left at the discretion of the treating surgeon. All surgeries were performed with the assistance of intraoperative imaging using a c-arm. At the end of the procedure standardized imaging was obtained with the c-arm (palmar-dorsal, lateral and skyline views) [15].

In general, functional aftercare with non-weightbearing free range of motion was allowed during the first six weeks after surgery. If the treating surgeon deemed it necessary, for example in very poor bone quality or when malcompliance was anticipated, a below the elbow cast was applied. Physical therapy was prescribed to all patients. Routine radiographic and functional follow-up was scheduled 6 weeks and 12 months after trauma.

### Baseline characteristics

Demographic data and treatment information was obtained from the electronic patient’s records. X-ray images were viewed and analyzed in the picture archiving and communication system (MERLIN). All fractures were classified according to the AO/OTA-classification [16] by two surgeons, disagreements were resolved by discussion with a third trauma surgeon. In case of open fractures, these were further classified according to the Gustilo classification [17]. The presence of a concomitant distal ulna fracture was noted.

### Outcomes

Surgery related variables included time from trauma to fixation, duration of surgery, number of screws/pegs used, fixation of ulna fracture (when applicable), and experience level of the surgeon or in case of a trainee performing the surgery the experience level of the supervising surgeon. The experience level was divided into three categories: junior attending (2–5 years after training completion), senior attending (5–10 years after training completion), and head of clinic (> 10 years after training completion). The postoperative treatment regime was assessed differentiating between functional aftercare (free range of movement as tolerated, non-weightbearing for 6 weeks) and cast-immobilization (4–6 week immobilization, followed by free range of movement and weightbearing as tolerated). The length of hospital stay was evaluated. In case of an outpatient operation the length of stay was defined as one day.

The intraoperative c-arm images were evaluated to determine the following: radial inclination, palmar tilt, ulnar variance, and Soong classification [18, 19]. An ulnar variance larger than zero meant the ulna was longer than the radius and vice versa. To assess for any loss of reduction the radial inclination, palmar tilt and ulnar variance were measured again on the 6-week and 12-month follow-up images. Loss of reduction was defined as a difference in radial inclination > 5 degrees, volar tilt > 10 degrees, and ulnar variance > 2 mm. As the ulnar variance differs depending on the rotation of the forearm, intra-operative dorso-palmar images had to be available to permit the comparison of ulnar variance with the 6-week and 12-month follow-up images. Secondary joint penetration by the implant was assessed. Time to fracture union was determined on the follow-up x-ray images. If the fracture line was no longer visible or bridging callus was present on three of the four cortices, fracture union was assumed [20].

Implants were not routinely removed. Implant-removal performed within 12 months after initial surgery or scheduled at the time of the 12-month follow-up was evaluated.

Complications that were assessed included infections, tendon ruptures and the occurrence of complex regional pain syndrome (CRPS). Infections will be defined based on the guidelines of The Centers for Disease Control and Prevention [21]. CRPS will be diagnosed according to the Budapest clinical diagnostic criteria [22].

### Statistical methods

The results were analyzed with the statistical software package SPSS Version 25. Descriptive statistics were provided for the baseline characteristics and study endpoints. Continuous variables were described as medians (with range) or means (with standard deviation) conditional on their distribution. Differences were analyzed using the Mann–Whitney-U-Test for non-normal data and the independent T-test for normally distributed data. Counts and percentages were calculated for categorical variables. Differences were assessed using the Fischer exact test. A p-value under 0.05 was considered significant.

## Results

### Participants

**Fig. 1:**
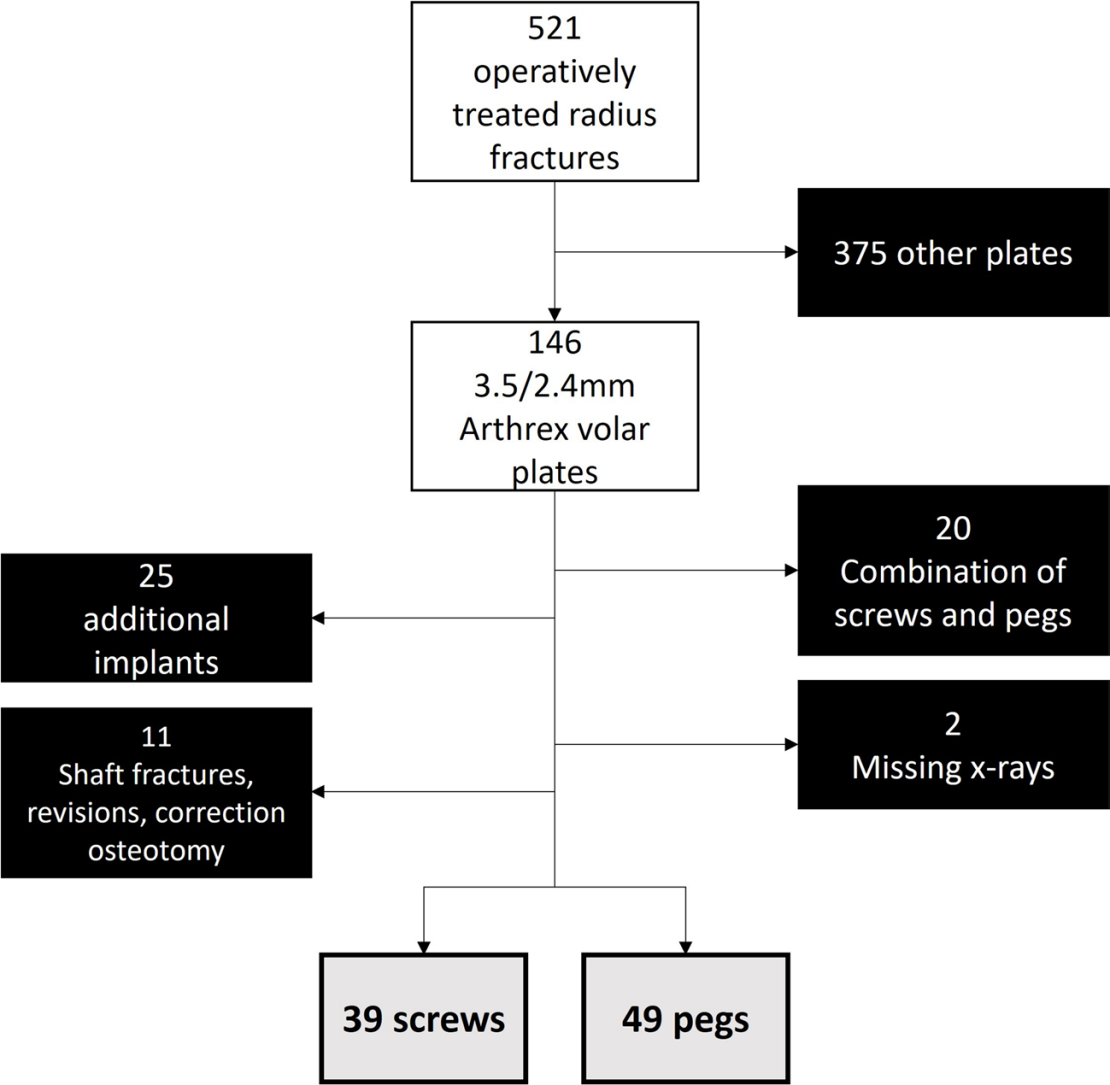
Flowchart of patient inclusion

Between June 2019 and May 2022 521 patients received operative treatment of a distal radius fracture at the study hospital. Of these patients, 146 were treated with the above-mentioned volar plate. After applying the exclusion criteria, a total of 88 patients could be ultimately included with 49 in the “peg group” and 39 in the “screw group” (Fig. 1).

The baseline characteristics of both groups can be found in Table 1. There were two significant differences between the groups. The first difference was that there were more smokers in the screw group (25.6% vs 8.2% *p* 0.04). The second difference was with regard to the AO-Classification of the fractures in each group, with more C-type fractures in the peg group and all B-type fractures having been treated with screws (*p* 0.04). Information regarding dexterity was missing in 39 of the patient files (11 in the screw group and 28 in the peg group).

**Table 1:** Baseline characteristics

		Screws (*n* = 39)	Pegs (*n* = 49)	*p*-Value
female		25 (64.1%)	33 (67.3%)	0.82
smoker		10 (25.6%)	4 (8.2%)	0.04
known osteoporosis		1 (2.6%)	3 (6.1%)	0.63
age at time of surgery		57.4 (SD 18.0)	58.6 (SD 19.9)	0.77
left hand injured		24 (61.5%)	35 (71.4%)	0.37
Dominant Hand (*n* = 49; 23 screw))		9 (39.1%)	10 (38.5%)	1
low energy trauma		15 (38.5%)	30 (61.2%)	0.053
temporary external fixator		4 (10.3%)	6 (12.2%)	1
Polytrauma		3 (7.7%)	3 (8.1%)	1
ASA classification				0.88
	I	9 (23.1%)	10 (20.4%)	
	II	25 (64.1%)	31 (63.3%)	
	III	5 (12.8%)	8 (16.3%)	
AO classification				0.04
	A	14 (35.9%)	14 (28.6%)	
	B	4 (10.3%)	0	
	C	21 (53.8%)	35 (71.4%)	
open fracture		1 (2.6%)	3 (6.1%)	0.4
ipsilateral distal ulna fracture		21 (53.8%)	30 (61.2%)	0.52
other Ipsilaterale arm injury		5 (12.8%)	4 (8.2%)	0.5

### Surgery related Data

All results regarding data related to the surgery can be found in Table 2. There were two significant differences between the groups. The mean duration of surgery was significantly longer in patients treated with screws (104 versus 81 min, p 0.003) and the experience level of the surgeon varied significantly between the groups (*p* < 0.01) with pegs being more frequently used when the head of clinic performed the surgery.

**Table 2:** Surgical outcomes

		Screws	Pegs	*p*-value
Mean days between trauma and surgery		5.8 (SD 4.3)	5.2 (SD 4.8)	0.56
Median duration of surgery in minutes (range)		104 (49–193)	81 (49–298)	0.003
Median number of pegs/screws in distal two rows (range)		5 (2–7)	6 (2–8)	0.9
Surgeon's experience level				0
	head of clinic	4 (10.3%)	26 (53.1%)	
	senior attending	17 (43.6%)	19 (38.8%)	
	junior attending	18 (46.2%)	4 (8.2%)	
fixation of ulna fracture		5 (12.8%)	9 (18.4%)	0.57
functional aftercare		30 (76.9%)	29 (59.2%)	0.1
length of hospital stay in days (range)		3 (1–31)	3 (1–27)	0.38

### Radiological outcome

Dorso-palmar intra-operative images were available in 62 patients and the lateral intra-operative view was missing in two patients. Table 3 shows the radiological results. Loss of reduction at the 6-week follow-up occurred in 5.9% of the patients who were treated with screws and 6.1% of those treated with pegs (*p* = 0.7). A subgroup analysis of only type C fractures demonstrated a loss of reduction at 6 weeks in one patient in each group (*p* = 1). A follow-up x-ray 12 months postoperatively was available for 50 patients of which 33 had intra-operative dorso-palmar images (11 from the screw group and 22 from the peg group). There was no significant difference in the occurrence of loss of reduction at 12 months between the groups (*p* = 1). The only significant difference regarding radiological variables was in the Soong classification with more Soong grade 1 (72.9% vs 47.4%, *p*-value 0.03) in the peg group.

**Table 3:** Radiological outcome

	Screws	Pegs	p-value
*Soong Classification (n* = *86)*	*n* = *38*	*n* = *48*	0.03
0	20 (52.5%)	13 (27.1%)	
1	18 (47.4%)	35 (72.9%)	
2	0	0	
*intraoperative measurements [mean (standard deviation)] (n* = *87)*			
radial inclination	17° (3.8)	18° (3.6)	0.6
palmar tilt (*n* = 86)	4.5° (4.99)	5.4 (3.9)	0.3
ulnar variance	- 1.6 mm (1.8)	−1.5 (2.6)	0.9
*6-week follow-up (n* = *83)*	*n* = *34*	*n* = *49*	
any loss of reduction	2 (5.9%)	3 (6.1%)	0.7
difference of radial inclination > 5°	1 (2.9%)	3 (6.1%)	0.6
difference of palmar tilt > 10°	0	0	-
difference of ulnar variance > 2 mm* (n = 62)	1 (5.3%)	0	0.3
12-month follow-up* (n = *33)*	*(n* = *11)*	(*n* = 22)	
any loss of reduction	1 (9.1%)	1 (4.5%)	1
joint penetration	0	1 (2%)	1

*only analyzed if intraoperative dorso-palmar images were available

Union was achieved by the time of the 12-month follow-up for all patients (*n* = 50) who had an x-ray at that time.

### Implant removal

Amongst all patients implant removal was performed in 22.7%. There was no significant difference in the rate of implant removals between the groups with 20.5% (8 patients) in the screw group and 24.5% (12 patients) in the peg group (p 0.8). The most commonly given reasons for implant removal were patient wish or volar irritation. In one case the removal was due to implant failure (broken screws), one patient (peg group) had a peri-implant fracture after a fall and one had a symptomatic instability of the distal radio-ulnar joint (screw group) which required revision including a correction osteotomy of the radius. In one case (peg group) removal was recommended to an asymptomatic patient based on his young age (19 years old).

### Complications

No infections or tendon ruptures occurred in either group. Three patients (3.4%) suffered from CRPS, with one in the screw group and two in the peg group (*p* = 1). One of the patients that experienced a loss of reduction and also exhibited joint penetration by a peg. Since the patient remained asymptomatic, no revision surgery or implant removal was necessary.

## Discussion

This retrospective cohort study comparing distal radius fractures treated with a volar plate either using angular stable screws or pegs in the distal fragment showed no difference with regard to loss of reduction at six weeks and 12 months after surgery. Union was achieved in both groups by the time of the 12-month follow-up and implant removal rates were similar.

Duration of surgery was significantly longer for patients who received screws, despite the pegs group having more complex fractures. However, these surgeries were more frequently performed or supervised by junior attendings, while most of the surgeries in the peg group were performed or supervised by a senior staff member. Senior staff members typically treat more complex cases and due to their experience, the duration of a surgery can be reduced. In the study hospital the head of clinic was familiar with the pegs while many junior attendings had not used them before. As the choice of implant was left at the discretion of the treating surgeon, this could explain the unequal distribution. It therefore seems unlikely that the use of pegs truly reduces operating time, instead this was probably due to the experience of the surgeons.

Previous biomechanical studies evaluating the stability of fracture fixation with either fixed angle screws or pegs have come to contradictory results. A study from 2008 examining axial loading of AO type C3 fractures in sawbones that were fixed with various combinations of screws and pegs showed earlier failure if the lunate fragment was solely stabilized with pegs [12]. Similarly, a study from 2010 evaluating AO type A3 fractures in sawbones fixed either using pegs or screws concluded that pegs were mechanically inferior under torsional loading [10]. In 2013 a biomechanical study using cadavers with simulated extraarticular fractures was unable to show a difference when axial loading was tested (this study group did not test torsional loading) [11]. A study from 2006 comparing the failure from axial loading of ten different plates with various fixation methods [23]. Due to the descriptive nature of the study no sound conclusion could be drawn. However, in their study, it seems that locking pegs may possibly be superior, as those plates only failed by means of plate bending with the pegs remaining firmly attached to the plate and undisplaced with the bone. The plates they were compared to included such with locking screws, cortical screws and locking tines, many of which are no longer available on the current market making the comparison difficult and possibly irrelevant today.

To the best of our knowledge only one previous clinical study has compared volar plating of distal radius fractures using angular stable pegs versus screws. Boretto et al. included 27 patients with AO type C2 or C3 fractures, of which 13 patients were treated with locking screws and 14 with locking pegs [13]. Their findings were in line with the findings of this current study. While some displacement occurred in both groups between the early and late postoperative x-rays, there was no difference in the delta value between the groups. While the current study did not examine each delta value, the number of patients that demonstrated secondary displacement was comparable in both groups. The aforementioned study performed an early postoperative and late x-ray. It is not clear when exactly these x-rays were obtained. The current study performed two comparisons, the first was between the intraoperative imaging and the x-ray at the six-week follow-up, the second was between the six-week and the 12-month follow-up. In addition, the current study assessed for displacement in both the ap- and lateral projections, while the previous study only assessed the ap-images. This could have uncovered a previously missed relevant displacement of the volar tilt, which however could not be detected, therefore supporting the conclusion Boretto et al. came to. This study enhances previous findings by including a larger number of patients and incorporating AO type A and B fractures, thereby making the results more generalizable.

Regarding joint penetration, no sound conclusion can be made based on this study. Joint penetration occurred in only one patient, making this complication too rare to provide any statistical value in this cohort.

With regards to plate positioning, there were more Soong 1 plates in the peg group than in the screw group. This could also be an effect of the complex fractures in the peg group, as these often require more distal placement of the plate to be able to support all fragments. Another possible explanation is that the more experienced surgeons that treated the peg patients, were less afraid of placing the plate to distal and risking joint penetration or soft tissue irritation. It seems unlikely that the choice of implant alone would influence the position of the plate. Contrary to previous literature, the Soong classification did not influence the rate of implant removal [24, 25].

The occurrence of CRPS was low in both groups with an overall occurrence of 3.4%. A recent meta-analysis demonstrated CRPS rates in patients with radius fracture to be as high as 13.63% [26]. According to the findings in that study, CRPS occurred more frequently in fractures with great complexity or associated soft tissue damage. Only 2.6% of the patients in the screw group and 6.1% in the peg group had open fractures (total 4 patients), which could explain the low rate of CRPS in this study cohort.

There are some limitations to this study that should be mentioned. Firstly, the retrospective study design prohibited randomization. Randomized control studies are often considered the gold standard when comparing treatment options. However, by leaving the choice of implant to the surgeons’ discretion, each surgeon was likely to choose the implant he/she is most comfortable and experienced with. This way differential expertise bias can be avoided and a sort of pseudorandomization occurs [27]. However, as within the study hospital, the head of clinic was most familiar with the locking pegs, this led to an imbalance in the distribution of fracture types with more complex fractures being treated with pegs. The fact that even though there were more complex fractures in the peg group, there were no differences in radiological outcome between the groups, only emphasizes the finding that pegs and screws are comparable with regard to radiological outcome. The question remains though, whether the outcome would have been the same, if the pegs were used by less experienced surgeons. Another limitation of observational studies is that data is often incomplete for some patients. This is an issue we encountered in our patients. Some required x-ray views were missing from the intraoperative images. As mentioned in the methods, ulnar variance could not be assessed in patients with missing dorso-palmar views. Despite this, these patients were included as all other measurements and outcomes were obtainable. It is possible that some of these patients may have had a loss of reduction based on the ulnar variance. However, excluding these patients from the study could have introduced further bias and reduced the patient cohort so much, that statistical analysis would have become impossible.

A further limitation is variability in the quality of postoperative x-ray images. Not all x-rays were ideal, which could lead to difficulties in measuring the variables accurately. The measurement accuracy is another limitation that should be mentioned. All measurements were performed by a single author. While this eliminates inter-observer variability, it could be possible that a systematic measuring mistake was overseen. As the outcome is based on the difference between two measurements at different times and not absolute numbers, the authors don’t believe this to be a relevant problem. Furthermore, the definitions for “loss of reduction” were chosen arbitrarily. Had different cut-off values been chosen, the results may have been different. Finally, the lack of functional outcome measures leaves it unknown, whether the loss of reduction caused any clinically relevant issues in any of the patients. An attempt was made to include functional outcome, however due to the retrospective design of the study, too much information was missing to be able to perform any evaluation.

In conclusion, this study showed locking pegs and screws to be comparable with regards to short and long-term radiological outcome in distal radius fractures treated with a volar plate. While in this study population surgery duration was shorter when pegs were used, this is most likely influenced by the treating surgeon’s experience level and not by the implant itself. Considering the potential benefits of smooth locking pegs, such as their blunt tips and possibly reduced risk of secondary joint penetration, pegs are a viable alternative to screws.

## Data Availability

No datasets were generated or analysed during the current study.
